# Increasing Mothers’ Confidence and Ability by Creating Opportunities for Parent Empowerment (COPE): A Randomized, Controlled Trial

**Published:** 2020

**Authors:** Reihaneh ASKARY KACHOOSANGY, Narges SHAFAROODI, Mohammad HEIDARZADEH, Mostafa QORBANI, Arash BORDBBR, Mahnaz HEJAZI SHIRMARD, Fatemeh DANESHJOO

**Affiliations:** 1 Department of Occupational Therapy, School of Rehabilitation, Shahid Beheshti University of Medical Sciences, Tehran, Iran; 2 Department of Occupational Therapy, School of Rehabilitation, Iran University of Medical Sciences, Tehran, Iran; 3 Department of Neonatology, Mashhad University of Medical Sciences, Mashhad, Iran.; 4 Department of Community Medicine School of Medicine, Alborz University of Medical Sciences Karaj, Iran; 5 Department of neonatology, Shahid Akbarabadi Hospital, Iran University of Medical Sciences, Tehran, Iran

**Keywords:** COPE, Intensive care, Neonates, Premature, Parental self-efficacy, Randomized controlled trial

## Abstract

**Objectives:**

Premature neonates are at great risk for cerebral palsy, developmental delays, hearing problems and visual impairments. Interventions to reduce the morbidities and adverse health outcomes in these neonates and improve parent-infant interaction are highly important. This study was conducted to determine the effect of the Creating Opportunities for Parent Empowerment (COPE) program on the perceived maternal parenting self-efficacy of premature parents.

**Materials & Methods:**

This was a randomized controlled trial with equal randomization (1:1:1 for 3 groups) and parallel group design. Forty-five preterm neonates were randomly allocated to treatment (n=15), supervision (n=15) and control (n=15) groups. COPE program was provided in the form of a 4-phase educational-behavioral intervention to the treatment and supervision groups. The primary outcome was parental self-efficacy, which was assessed by the Perceived Maternal Parenting Self-Efficacy inventory**.** All the measurements were performed pre- and post-completion with the valid equipment and by blind assessors.

**Results:**

COPE mothers reported significantly stronger beliefs regarding their parental role and have more confidence to their ability in caring of neonates compared with control mothers (P-value <0.001).

**Conclusion:**

An educational-behavioral intervention would strengthen mothers’ belief in themselves and knowledge about their neonates and would enhance premature mothers’ ability to care for their neonates as well as parent-infant interaction.

## Introduction

The birth of a healthy and normal neonate is a celebrated event with happiness for parents, but this is different when a neonate is born prematurely and parents face an unexpected event that involves many challenges (1, 2). Parents experience high levels of stress as a result of the preterm birth, diagnosis and hospitalization of neonates that is characterized by sense of fear, guilt, sadness, hostility, irritability, grief, helplessness and loss of confidence (3). 

Parents’ challenges revolve around issues such as learning to care for the newborn, obtaining information about the neonate, getting to know the baby and dealing with one’s own expectations as a parent (4). A review of the available literature demonstrated that parents usually feel incompetent in caring for their neonate after her/his discharge from the neonatal intensive care unit (NICU). Indeed, self-efficacy, that is parents’ perception of themselves as a capable and effective person in assuming parental role (5), is at low level, indicating the importance of training in this population group (6). 

There is considerable scientific evidence supporting the significance and necessity of increasing self-efficacy in parents (7), because the degree of self-efficacy is effective in the quality of the care provided by parents as well as the degree of parents’ satisfaction with their parenting experience (8). Boosting parents’ self-efficacy is important in coping with stress in NICU (9). Also, perceived competence in the parenting role is directly associated with emotional and parental behaviors, the quality of newborn care and infant’s future developmental outcomes (7, 10).

Premature parents need to feel efficacious in their parenting role, and parents' practical training and strategies for increasing parents and families’ involvement in neonatal care (work book) have important consequences for parent and infant development (11).

Creating Opportunities for Parent Empowerment (COPE) is an educational-behavioral intervention designed based on the presumption that providing parental supportive interventions benefits parents, neonates and families in general (12-14). A review of the literature revealed that COPE can lower the stress level through increasing parents’ knowledge and modifying their beliefs about their preterm neonate (15, 16).

Most studies on the efficacy of COPE conducted in different regions of the United States are mainly focused on the mental (i.e., anxiety, depression, negative emotion, and participation in caring) and physical health of parents and their infants (weight gain) (16). Also, there is strong evidence that parent training enhances maternal self-efficacy and empowers mothers for breastfeeding (17, 18).

However, there is a gap in the literature concerning how parents’ self-efficacy is associated with caring for a preterm infant at home, especially during the first weeks and months after hospital discharge. The purpose of this study was to evaluate the effect of the COPE program on the perceived maternal parenting self-efficacy (PMP-SE) of premature parents.

## Materials & Methods

This was a double-blind, randomized, controlled trial conducted in the two university hospitals of Akbar-abadi and Mahdieh in Tehran, Iran, during March 2015 to September 2015. We included 45 neonates with a gestational age (GA) of under 37 weeks who were admitted to the NICUs of the two hospitals. These preterm neonates met the following inclusion criteria: (1) birthweight between 1000 and 2500 gr, (2) GA under 37 weeks, (3) 5-minute Apgar score of 7 and more, (4) no major abnormalities on brain ultrasound (grade III or IV intraventricular hemorrhage [IVH]), (5) absence of congenital anomalies or neuromuscular disorders and (6) hospitalization in NICU for at least 7 to 30 days. The exclusion criteria comprised of incurable disease, neonatal death during the study and parents’ unwillingness to participate in the study for any reason.

Sample size was calculated with an α-value of 5% and power of 80%. The analysis accounted for a 20% attrition rate. Fifteen neonates per group were needed to detect clinically worthwhile effects.

After obtaining free and informed consent, neonate randomization was performed using a randomized block design, and the neonates were randomly assigned to the supervision (n=15), treatment (n=15) and control (n=15) groups. After gathering the clinical data, mothers were asked to rate their perceptions about their ability to effectively and successfully assume their caring role as a mother. PMP-SE was measured using the 20-item Efficacy subscale of the Parenting Sense of Competence scale (17). Items were rated on a 4-point Likert scale ranging from “strongly agree” to “strongly disagree.”

Neonates in the control group received no additional treatment from the research therapist, but they received the routine interventions and services, and the COPE program was provided for the treatment and supervision groups. This educational-behavioral intervention program consisted of a series of CDs along with written information and reinforcing activities for parents (workbook). COPE program was delivered in four phases as follows: phase I: 2-4 days after the infant was admitted to the NICU, phase II: 2-4 days after the first phase, phase III: 1-4 days prior to the infant’s NICU discharge, and phase IV: about one week after discharge. 

**Fig. 1:**
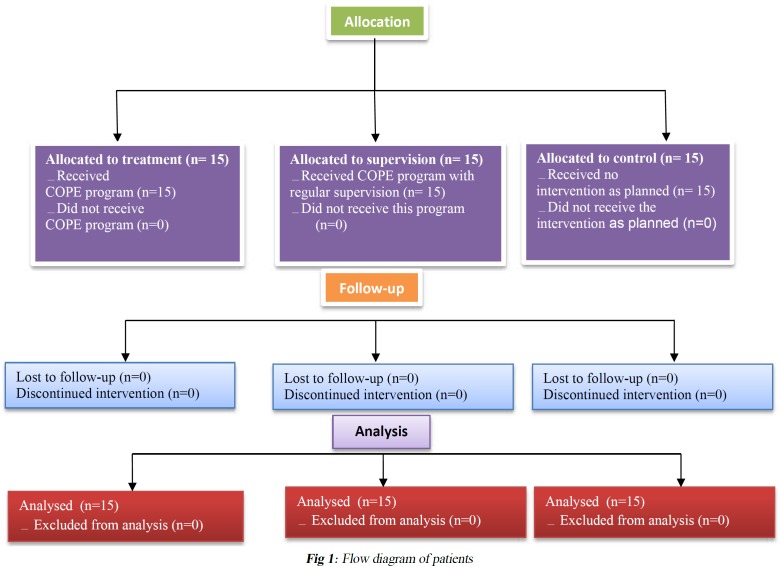
Flow diagram of patients

Daily strict implementation of the program was followed by the therapist in the supervision group; the therapist also provided daily comments and reviews. Follow-up assessments were completed one month after discharge by the same person, who was unaware of the group allocations (Figure 1). The assessor was a master of Occupational Therapy who was expert in the field of pediatric family-centered occupational therapy with four years of experience inneonatal therapy.

The data were analyzed using SPSS. All the values were tabulated as averages (mean) with standard deviation (SD). For all the analyses, the significance level was set at 0.05.

## Results

The mean gestational age of the premature infants was 31.93 weeks in the control group (SD: 2.71; range: 26–36 weeks), 33.26 weeks in the treatment group (SD: 1.03; range: 32-35 weeks) and 33.26 weeks in the supervision group (SD: 2.08; range: 28-34 weeks). As shown in Table 1, neonates in all the three groups were matched for all the parameters (i.e., gestational age, birth weight, birth length, head circumference at birth, Apgar scores [1 and 5 min], and prematurity), and there were no significant differences between them at baseline. According to the results (Table 2), the mean PMP S-E scores of the mothers in the treatment and supervision groups were higher than in the control group, and there was a significant difference between the three groups (*P* = 0.000). 

**Table 1:** Participants’ socio-demographics at baseline

**variable**	**Control group** **Mean ± SD**	**Treatment group** **Mean ± SD**	**Supervision group** **Mean ± SD**	**P-value**
**Birth Weight**	1594±476.10	1854.66±337.88	1752.66±351.63	0.195
**Head Circumference**	29.63±2.22	30.40±0.98	29.96±1.88	0.50
**Birth Length**	41.40±3.96	42.66±3.84	43.93±3.30	0.18
**Apgar 1** ^st^ ** min**	8.33±0.81	8.33±0.72	8.41±0.79	0.95
**Apgar 5** ^th^ ** min**	9.33±0.81	9.46±0.63	9.58±0.90	0.71
**Gestational Age**	31.93±2.71	33.26±1.03	33.26±2.08	0.19

**Table 2:** Comparison of PMP S-E in the treatment, supervision and control groups

	**Treatment group** **Mean ± SD**	**Supervision group** **Mean ± SD**	**Control group** **Mean ± SD**	**P-value**
**Pre-intervention**	24.80 ± 2.30	23.13 ± 1.30	23.66 ± 2.16	0.000^*^
**Post-intervention**	54.53 ± 6.25	56.46 ± 4.77	45.40 ± 3.35	

* Univariate Analysis of Variance

## Discussion

Evidence shows that insufficient information and knowledge about neonatal care is a concerning factor for mothers in the postpartum period (19, 20). According to the results, training for mothers had a significant positive impact on maternal self-efficacy. COPE program promoted self-efficacy score in both treatment and supervision groups compared to the control group. This finding is in line with those of a systematic review by Brett et al. (2011) in that evidence indicate that developmental and behavioral care programs such as COPE significantly increase mothers’ knowledge of their neonates’ needs and conditions and improve mothers’ confidence and ability to care for their neonates (19). Brett’s study showed that interventions for providing support to parents are sectional and delivered in one of the following four stages: A-prenatal, B- NICU, C- discharge or D- home. However, the present study entailed regular behavioral and practical support from hospital to home.

Findings of the present study showed that training programs for mothers had a positive impact on mothers’ perception of their neonates’ behaviors and they also improved mothers’ monitoring strategies versus the control group. The present findings are consistent with the results obtained in a meta-analysis by Letarte et al. (2010). They reported that parents training had a positive effect on parents’ self-efficacy and improved parent-child relationship (21). Parent training aims to modify parents’ beliefs to promote child’s development. More specifically, the goals of these programs are to encourage parents to increase their sensitivity to the infant and develop appropriate managing and problem-solving skills. 

A literature review indicated that traditional office-based family therapy services were not always useful and effective in high-risk families (22). Also, training with supervision can develop trainee’s knowledge and skills through organizing, implementing and providing feedback. In this study, the comparison between supervision and treatment groups showed no significant difference (P-value = 0.121), and this finding is inconsistent with the available evidence. This finding could be attributed to the hard effort and active participation of parents to learn more, adapt to the conditions and reduce the complications of prematurity in the golden neonatal period; thus, they meticulously applied all the learned skills. These findings suggest that training programs can strengthen mothers’ beliefs and knowledge about their neonates and enhance their neonatal care skills and parent-infant interaction.

## Limitations

The limitation of this study included limited sample size; thus, we recommend more comprehensive clinical trials on the effect of parent training on developmental outcomes in preterm infants.

## In Conclusion

Parent training programs have been found to be very effective as most parents need careful training and support to improve their parenting skills. Thus, the present study emphasizes the need to consider learning opportunities and delivering parent training packages in hospitals and after discharge. 
